# A Formal Language-Based Approach in Biology

**DOI:** 10.1002/cfg.364

**Published:** 2004-02

**Authors:** Marian Gheorghe, Victor Mitrana

**Affiliations:** 1 Department of Computer Science University of Sheffield Sheffield S1 4DP UK; 2 Research Group in Mathematical Linguistics Rovira i Virgili University Pça Imperial Tàrraco 1 Tarragona 43005 Spain

## Abstract

This paper presents an overview of computational biology approaches and surveys some of the natural computing models using, in both cases, a formal language-based approach.


**Comparative and Functional Genomics**
*Comp Funct Genom* 2004; **5**: 91–94.
Published online in Wiley InterScience (www.interscience.wiley.com). **DOI:** 10.1002/cfg.364

**Conference Review**

### A formal language-based approach in biology

Marian Gheorghe[1]* and Victor Mitrana[2]

[1] *Department of Computer Science, University of Sheffield, Sheffield S1 4DP, UK*
[2] *Research Group in Mathematical Linguistics, Rovira i Virgili University, Pça, Imperial Tàrraco 1, 43005 Tarragona, Spain*

**Correspondence to: Marian Gheorghe, Department of Computer Science, University of Sheffield, Sheffield S1 4DP, UK. E-mail: m.gheorghe@dcs.shef.ac.uk*

Received: 12 November 2003
Revised: 18 November 2003
Accepted: 25 November 2003

### Abstract

**This paper presents an overview of computational biology approaches and surveys some of the natural computing models using, in both cases, a formal language-based approach.** **Copyright © 2004 John Wiley & Sons, Ltd.**

**Keywords: Chomsky hierarchy; Lindenmayer systems; DNA computation; gene (un)scrambling in ciliates; P-systems**

### Introduction

The scope of 'computational biology' covers mostly all aspects of computational modelling (including mathematical models) that refer to biology, especially molecular biology. The International Society for Computational Biology (**http://www.iscb.org**) emphasizes 'the role of computing and informatics in advancing molecular biology'. The models that can be used cover a wide spectrum: continuous and discrete-based approaches, deterministic vs. stochastic ones, and integrative complex hybrid methods. The range of discrete models used in biology contains Petri nets, CCS, formal languages, discrete probabilistic approaches, and so forth.

The so called 'modern era' of both formal language theory and biology started approximately in the same period, the 1950s, when Noam Chomsky introduced the concept of formal grammar [6] (a revolutionary approach in linguistics, which further on led to the well-established field of formal language theory) and Watson and Crick discovered the double helical structure of the DNA molecule [40]. Chomsky's hierarchy of languages has been intensively studied for its formal properties [30], but also led to important applications, not only in computational linguistics [7] but also in syntactic pattern processing, speech recognition [21], programming language syntax, etc. Although biologists have long made use of linguistic metaphors in describing processes involving nucleic acids, protein sequences and cellular phenomena, since the 1980s molecular sequences started to be investigated with the methods and tools derived from Chomsky's legacy [14,33–35]. Some surveys present various applications of Chomsky grammars or derivatives of them in biology [36,37]. On the other hand, computational biology led, in the context of formal language-based modelling, to the emergent area of natural computing, which investigates new computational paradigms rooted in biology [28].

This review presents an overview of computational biology and natural computing, using in both cases a formal language-based approach.

### Computational biology and formal languages

Around the 1970s and even before this date, formal grammars had been considered as models of

Copyright © 2004 John Wiley & Sons, Ltd.

heredity. Pawlak [26] used dependency grammars as an approach in the study of protein formation. In this approach sequences of codons formed by three nucleotides were considered as the basic constituents of proteins, and associated strings of codons with proteins. For similar purposes, Marcus [24] considered so-called semi-Lindenmayer systems; moreover, an isomorphism between the genetic and natural language was also discussed.

Treating chromosomes and genomes as languages raises the possibility that the structural information contained in biological sequences can be generalized and investigated by formal language theory methods. A pioneering work describes very simple genes by means of regular grammars, although different features of nucleic acids cannot be modelled by regular expressions [4]. Other biological phenomena, such as gene structure and expression [34] and conformation of macromolecules [33], are modelled by using formal grammars. Generative grammars have been used to construct an integrative paradigm of the organization and regulation of gene expression [8,9]. The application of a phrase-structure grammar is justified by the existence of lexical categories. Four transformational rules applied in accordance with two principles are used to represent loops of regulation. Specifically, such a grammar generates all and only those arrays that are consistent with the principles of the system of regulation of $\sigma^{70}$ promoters in *Escherichia coli* [10]. This grammatical model is naturally extended to include qualitative dynamic descriptions of the operons [11].

Stochastic context-free grammars constructed from sample sets of sequences were considered in order to model RNA sequences [18,31]. Definite clause grammars and cut grammars were used to investigate the gene structure and expression of various DNA sequences or different forms of chromosomal mutation and rearrangement [34,35]. It is suggested [35] that both the syntactical and the functional structure of formal grammars can be modelled by sets of nucleotides and hybridization experiments, respectively.

Based on Chomsky's hierarchy, and Kolmogorov and Chaitin complexity concepts, a grammatical approach to the syntactical analysis of polypeptides and polynucleotides has been developed [15]. It is shown that the derivational length has a tendency to increase along phylogenetic pathways.

Yokomori and Kobayashi use local language learning and its applications on an important combinatorial problem: sequence analysis [42]. Furthermore, haemoglobin amino acid sequences are modelled by means of local automata. A formal language theoretical framework is employed to investigate the primitive constructs that are minimally required for obtaining a genetic language of a certain complexity [41]. Further, this work, as well as another study [39], shows that another grammar formalism, that of tree-adjoining grammars (which is intensively investigated in mathematical linguistics), has great potential to predict various RNA structures (loop, pseudo-knot, etc.) for different biological data.

A computational model, similar in a certain sense to a generative grammar since it is based on multiset rewriting, is used to simulate the emergence of autocatalytic cycles, which are often found in living systems [38]. The use of X-machines, a variant of finite state machines with much more computational power, is used to model immunological pathways [20].

Similarities between cellular processes or DNA sequences and textual and literary approaches are investigated in order to deepen our understanding of some fundamental issues of the nature of bioinformation [37,25]. Some studies involve statistical analysis at the level of vocabularies similar to those of comparative linguistics [5,29].

### Natural computing models

Although the idea of natural computing is relatively new [28], it may be traced back to the first generative models of simple algal growth. The idea of sequential rewriting used in the theory of formal languages was very productively modified for the purposes of describing such processes. A complete parallel rewriting paradigm was introduced by Lindenmayer [23] in order to model the growth process. From the biological point of view, it cannot be expected that the components of any biological organism evolve sequentially or that cell reproduction may be modelled within a sequential approach. It is more likely that the cells that can reproduce simultaneously would be modelled by a mechanism that behaves the same. In the theory of L systems, a colony of biological cells is represented by a string

Copyright © 2004 John Wiley & Sons, Ltd.

of symbols: for each individual cell one appearance of a symbol is associated with it, and different states of cells are represented by different symbols. Cell state changes are modelled by rewriting rules replacing each symbol by another symbol or by several symbols (in the case of reproduction), as in formal grammars. The parallel nature of the cell state changes and cell division is modelled by the parallel execution of the symbols rewriting according to the rules applied.

The fundamental mechanism by which genetic material is merged is called 'recombination' — DNA sequences are recombined under the effect of enzymatic activities. Head [19] introduced the *splicing* operation as a language theoretical approach of the recombinant behaviour of DNA sequences under the influence of restriction enzymes and ligases. According to this approach, a splicing operation consists of cutting two DNA sequences at specified sites, and then the first substring of one sequence and the second segment of the other are linked at their sticky ends, and vice versa. A new type of computability model called H systems, based on the splicing operations, has been considered. Many variants of H systems have been invented and investigated (regulated H systems, distributed H systems, H systems with multisets, etc.). Under certain circumstances, the H systems are computationally complete and universal [30]. These results suggest the possibility of considering H systems as theoretical models of programmable universal DNA computers based on the splicing operation.

The bio-operations of gene (un)scrambling in ciliates have been considered as formal operations on strings and languages. First, a computational model based on one intermolecular and one intramolecular operation has been considered [22]. Another model suggested by the intricate process that transforms the DNA in the micronucleus of some hypotrichous ciliates into that of the macronucleus, based upon three intra-molecular operations, has been devised [16] and then investigated for various properties. Many other transformations, besides DNA unscrambling, are part of the global process of transforming the micronucleus into the macronucleus that led to the concept of macronuclear strings and language [17].

Chromosomal rearrangements, including pericentric and paracentric inversions, intrachromosomal and interchromosomal transpositions, and translocations are modelled as operations on languages [12,32,41]. A language-generating mechanism based on the operations suggested by all of the mutations mentioned above is introduced [13] and some properties studied [2].

A supercell system (also called P system) is a theoretical model of the behaviour and functioning of the cell membrane, which serves as an interface between various inner layers and the cell interior and the exterior environment, within a multicellular structure [27]. P systems are distributed parallel computing devices of a biochemical inspiration, incorporating complex chemical reactions involving various molecules, catalysts, promoters or inhibitors, electrical charges, etc. and borrowing ideas from Lindenmayer systems, grammar systems, chemical abstract machines and multisets rewriting. A sound theory of P systems and P algorithms has been emerging during recent years. Simulations of P systems as X machines or communicating X machines [1] and investigations into combining P systems with X-machines as a tissue theoretical model have been reported recently [3].

### Acknowledgement

The work of the first author has been supported by EPSRC grant GR/R84221/01.

### References

1. Aguado J, Bălănescu T, Cowling T, *et al.* 2002. P systems with replicated rewriting and stream X machines. *Fund Inform* **49**: 17–33.
2. Ardelean I, Gheorghe M, Martin-Vide C, Mitrana V. 2003. A computational model of cell differentiation. In *Pre-Proceedings of the Fifth International Workshop on Information Processing in Cells and Tissues*, Lausanne; 275–287.
3. Bernardini F, Gheorghe M, Holcombe M. 2003. PX systems = P systems + X machines. *Natural Comput* **2**: 201–213.
4. Brendel V, Busse HG. 1984. Genome structure described by formal languages. *Nucleic Acids Res* **12**: 2561–2568.
5. Brendel V, Beckmann JS, Trifinov EN. 1986. Linguistics of nucleotide sequences: morphology and comparison of vocabularies. *J Biomol Struct Dynam* **4**: 11–21.
6. Chomsky N. 1957. *Syntactic Structures*. Mouton: The Hague.
7. Cole RA, Mariani J, Uszkoreit H, Zaenen A, Zue V. 1997. *Survey of the State of the Art in Human Language Technology*. National Science Foundation, European Commission. Cambridge University Press: Cambridge.
8. Collado-Vides J. 1989. A transformational-grammar approach to the study of the regulation of gene expression. *J Theoret Biol* **136**: 403–425.
9. Collado-Vides J. 1991. The search for grammatical theory of gene regulations is formally justified by showing the

Copyright © 2004 John Wiley & Sons, Ltd. 

inadequacy of context-free grammars. *Comput Appl Biosci* **7**: 321–326.
10. Collado-Vides J. 1992. Grammatical model of the regulation of gene expression. *Proc Natl Acad Sci USA* **89**: 9405–9409.
11. Collado-Vides J, Gutiérez-Rios RM, Bel-Enguix G. 1998. Networks of transformational regulation encoded in a grammatical model. *BioSystems* **47**: 103–118.
12. Dassow J, Mitrana V. 1997. On some operations suggested by genome evolution. In *Proceedings of the Second Pacific Symposium on Biocomputing*, Altman RB, Danker AK, Hunter L, Klein TE (eds). 97–108.
13. Dassow J, Mitrana V, Salomaa A. 1997. Context-free evolutionary grammars and the structural language of nucleic acids. *BioSystems* **43**: 169–177.
14. Dong S, Searls DB. 1994. Gene structure prediction by linguistic methods. *Genomics* **23**: 540–551.
15. Ebeling W, Jimenez-Montano MA, 1980. On grammars, complexity and information measures of biological macromolecules. *Math Biosci* **52**: 53–71.
16. Ehrenfeucht A, Prescott D, Rozenberg G. 2001. Computational aspects of gene (un)scrambling in ciliates. In *Evolution as Computation*, Landweber L, Winfree E (eds). Springer-Verlag: Berlin; 45–86.
17. Freund R, Martin-Vide C, Mitrana V. 2002. On some operations suggested by gene assembly in ciliates. *New Generat Comput* **20**: 279–293.
18. Grate L, Herbster M, Hughey R, *et al*. 1994. RNA modelling using Gibbs sampling and stochastic context-free grammars. In *Proceedings of the Second International Conference on Intelligent Systems for Molecular Biology*. AAAI/MIT Press: Menlo Park, CA.
19. Head T. 1987. Formal language theory and DNA: an analysis of the generative capacity of specific recombinant behaviours. *Bull Math Biol* **49**: 737–759.
20. Holcombe M, Bell A. 1998. Computational models of immunological pathways. In *Information Processing in Cells and Tissues*, Holcombe M, Paton R (eds). Plenum: New York; 213–226.
21. Jurafsky D, Martin JH. 2000. *Speech Language Processing*. Prentice Hall: New York.
22. Kari L, Landweber L. 2000. Computational power of gene rearrangements. DNA5, DIMACS series in Discrete Mathematics and Theoretical Computer Science, Winfree E, Gifford D (eds). *Am Math Soc* **54**: 207–216.
23. Lindenmayer A. 1968. Mathematical models of cellular interactions in development. I. Filaments with one-sided inputs. II. Simple and branching filaments with two-sided inputs. *J Theoret Biol* **18**: 280–299, 300–315.
24. Marcus S. 1974. Linguistic structures and generative devices in molecular genetics. *Cahiers Ling Theor Appl* **1**: 77–104.
25. Paton R, Matsuno K. 1998. Verbs, glue and categories in the cellular economy. In *Information Processing in Cells and Tissues*, Holcombe M, Paton R (eds). Plenum: New York; 253–260.
26. Pawlak Z. 1965. *Grammars and Mathematics*. Panstwowe Zakady Wydawnietw Szkolnych: Warzsawa (in Polish).
27. Păun G. 2002. *Membrane Computing. An Introduction*. Springer-Verlag: Berlin.
28. Păun G, Rozenberg G, Salomaa A. 1998. *DNA Computing. New Computing Paradigms*. Springer-Verlag: Berlin.
29. Pevzner PA, Borodovsky MY, Mironov AA. 1989. Linguistics of nucleotide sequences I, II. *J Biomol Struct Dyn* **6**: 1013–1038.
30. Rozenberg G, Salomaa A (eds). 1997. *Handbook of Formal Languages*. Springer-Verlag: Berlin.
31. Sakakibara Y, Brown M, Hughey R, *et al*. 1994. Stochastic context-free grammars for tRNA modelling. *Nucleic Acids Res* **25**: 5112–5120.
32. Searls DB. 1988. Representing genetic information with formal grammars. Proceedings of the 7th National Conference on Artificial Intelligence. *Am Assoc Artif Intell* **7**: 386–391.
33. Searls DB. 1992. The linguistics of DNA. *Am Scient* **80**: 579–591.
34. Searls DB. 1993. The computational linguistics of biological sequences. In *Artificial Intelligence and Molecular Biology*, Hunter L (ed.). AAAI/MIT Press: Menlo Park, CA; 47–120.
35. Searls DB. 1995. Formal grammars for intermolecular structure. In *IEEE Symposium on Intelligence in Neural and Biological Systems*. IEEE Computer Society Press: Hernolen, Virginia; 30–37.
36. Searls DB. 1997. Linguistic approaches to biological sequences. *Bioinformatics* **13**: 333–344.
37. Searls DB. 2002. The language of genes. *Nature* **420**: 211–217.
38. Suzuki Y, Tanaka H. 1997. Symbolic chemical system based on abstract rewriting system and its behavior pattern. *Artif Life Robotics* **1**: 211–219.
39. Uemura Y, Hasegawa A, Kobayashi S, Yokomori T. 1999. Tree adjoining grammars for RNA structure prediction. *Theoret Comput Sci* **210**: 277–303.
40. Watson JD, Crick FHC. 1953. Molecular structure of nucleic acids. *Nature* **171**: 737–738.
41. Yokomori T, Kobayashi S. 1995. DNA evolutionary linguistics and RNA structure modelling: a computational approach. In *IEEE Symposium on Intelligence in Neural and Biological Systems*. IEEE Computer Society Press: Hernolen, Virginia; 38–45.
42. Yokomori T, Kobayashi S. 1998. Learning local languages and their application to DNA sequence analysis. *IEEE Trans Pattern Machine Intell* **20**: 1067–1079.

Copyright © 2004 John Wiley & Sons, Ltd. 
